# Trajectories of Work-to-family Conflict and Depressive Symptoms in Hong Kong: A 15-year Prospective, Population-based Cohort Study

**DOI:** 10.1192/j.eurpsy.2026.10463

**Published:** 2026-07-31

**Authors:** R. Long, C. S. Wong, W. W. Mak, S. Manuo, M. Y. Ni, Y. Ni

**Affiliations:** 1 School of Public Health, The University of Hong Kong; 2 Department of Psychology, The Chinese University of Hong Kong, Hong Kong, SAR, China; 3 Faculty of Social Sciences, Tampere University, Tampere, Finland; 4 The University of Hong Kong, Hong Kong, SAR, China

## Abstract

**Introduction:**

Work-family conflict is a prominent and rapidly growing stressor affecting a substantial portion of the working population. Although research on its relationship with mental health is expanding, important gaps remain, particularly regarding the long-term trajectories of work-to-family conflict (WFC) and its impact on mental health outcomes.

**Objectives:**

To identify distinct longitudinal trajectories of WFC over 15 years and to evaluate how the longitudinal associations between WFC and depressive symptoms differ across these latent classes.

**Methods:**

Participants were drawn from the FAMILY Cohort, assessed at three waves: 2009–11 (Wave 1, n=11,242), 2011–14 (Wave 2, n=6,056), and 2023–24 (Wave 3, n=1,061). WFC was measured using the 6-item WFC Scale, and depressive symptoms were assessed using the Patient Health Questionnaire-9 (PHQ-9). The analytical sample consisted of participants with complete data on both measures across all three waves (N=791). To adjust for potential selection bias, inverse probability weighting and raking based on Hong Kong census demographics were applied. Growth Mixture Modeling (GMM) was performed to identify distinct trajectories of WFC. A two-level generalized linear mixed model (GLMM) was applied to evaluate the longitudinal associations between WFC and depressive symptom scores within each WFC trajectory. The model included a random intercept and a quadratic term for time, and was adjusted for baseline socio-demographic, family, and work characteristics. All analyses were conducted using Mplus version 8.3.

**Results:**

GMM identified three distinct longitudinal trajectories of WFC (Figure A): (1) a high and escalating trajectory (27%), (2) a moderate and stable trajectory (31%), and (3) a low and declining trajectory (42%). Figure B indicates a nonlinear change in the mean depressive symptoms scores across all groups. The GLMM model identified a positive within-person association between WFC and depressive symptoms across all groups. The strength of this association varied by trajectory: the high and escalating group (IRR = 1.25, 95% CI [1.15, 1.37]), the low and declining group (IRR = 1.15, 95% CI [1.08, 1.24]), and the moderate and stable group (IRR = 1.12, 95% CI [1.03, 1.22]).

**Image 1:**
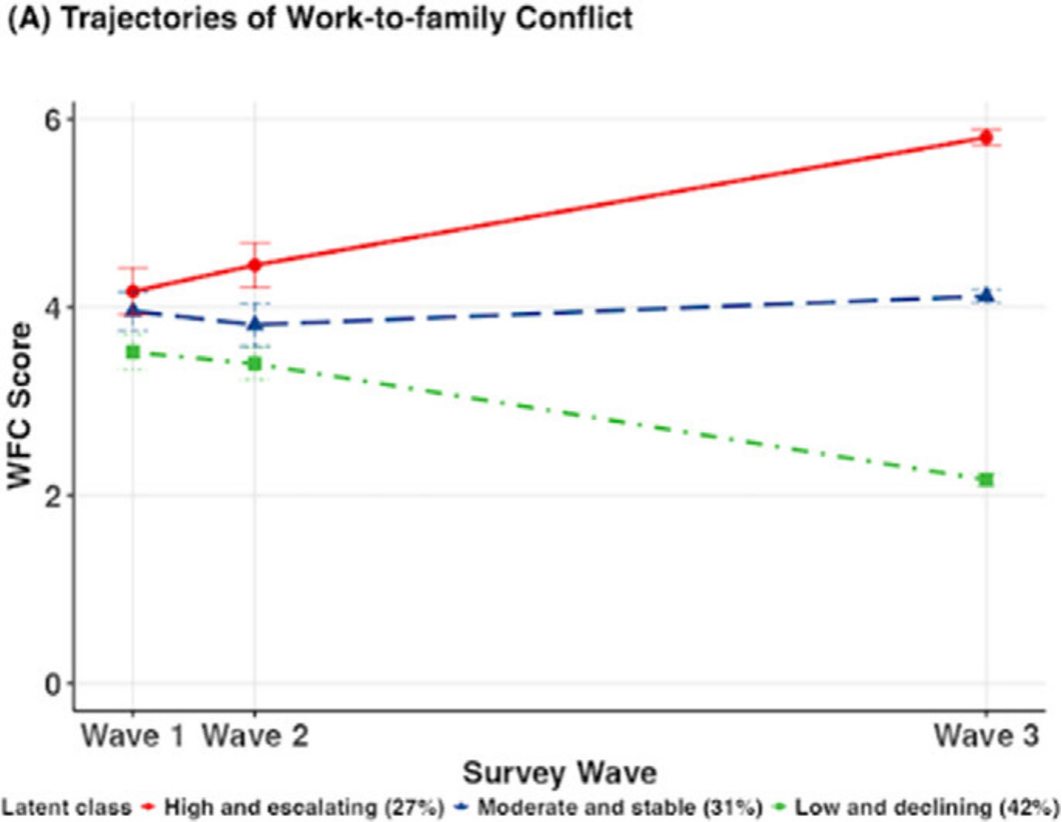
Image 1: Long description.

**Image 2:**
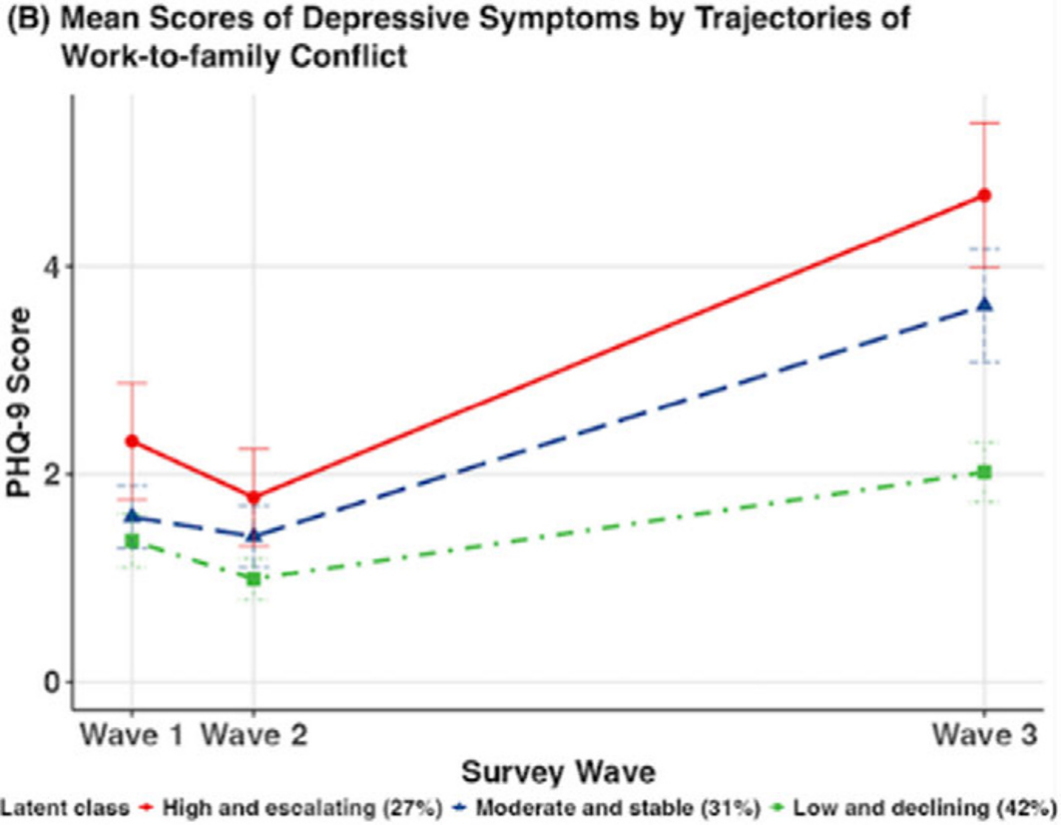
Image 2: Long description.

**Conclusions:**

This longitudinal study provides critical insights into the heterogeneous nature of WFC trajectories and their impacts on mental health over a 15-year period. By identifying three distinct WFC trajectories, about 27% of the working population experiences chronic, high-intensity WFC. A consistently positive within-person association between WFC and depressive symptoms was observed across all WFC trajectory groups, suggesting that while persistent high exposure to WFC may pose a greater risk, individuals in lower-intensity trajectories remain vulnerable to its mental health consequences.

**Disclosure of Interest:**

None Declared

## Long descriptions

### Image 1: Long description

A line graph titled A Trajectories of Work-to-family Conflict. The Y-axis is labeled W F C Score and ranges from 0 to 6 in increments of 2. The X-axis is labeled Survey Wave with three points: Wave 1, Wave 2, and Wave 3. Three latent class trajectories are plotted with error bars.

* High and escalating (27 percent): Represented by a solid red line with circular markers. It starts at a score of approximately 4.2 at Wave 1, increases slightly to 4.5 at Wave 2, and rises sharply to nearly 6.0 at Wave 3.

* Moderate and stable (31 percent): Represented by a dashed blue line with triangular markers. It starts at 4.0 at Wave 1, dips slightly to 3.8 at Wave 2, and returns to approximately 4.1 at Wave 3.

* Low and declining (42 percent): Represented by a dash-dot green line with square markers. It starts at 3.5 at Wave 1, remains stable at 3.4 at Wave 2, and declines significantly to approximately 2.2 at Wave 3.

Navigate back to Image 1:.

### Image 2: Long description

The X axis is labeled Survey Wave with three points: Wave 1, Wave 2, and Wave 3. The Y axis is labeled P H Q 9 Score with a scale from 0 to 4. The graph tracks three latent classes:

* High and escalating (27 percent): Represented by a solid red line with circular markers. It starts at a score of approximately 2.3 at Wave 1, dips slightly to 1.8 at Wave 2, and then shows a steep linear increase to approximately 4.8 at Wave 3.

* Moderate and stable (31 percent): Represented by a dashed blue line with triangular markers. It starts at 1.6 at Wave 1, dips to 1.4 at Wave 2, and increases to approximately 3.6 at Wave 3.

* Low and declining (42 percent): Represented by a dash-dot green line with square markers. It starts at 1.4 at Wave 1, dips to 1.0 at Wave 2, and rises to 2.0 at Wave 3.

All data points include vertical error bars indicating confidence intervals. The overall trend shows that while all groups experienced a slight decrease between Wave 1 and Wave 2, they all saw an increase in depressive symptoms by Wave 3, with the High and escalating group showing the most significant rise.

Navigate back to Image 2:.

